# Searching for New Tools to Counteract the *Helicobacter pylori* Resistance: The Positive Action of Resveratrol Derivatives

**DOI:** 10.3390/antibiotics9120891

**Published:** 2020-12-10

**Authors:** Paola Di Fermo, Silvia Di Lodovico, Rosa Amoroso, Barbara De Filippis, Simonetta D’Ercole, Emanuela Di Campli, Luigina Cellini, Mara Di Giulio

**Affiliations:** 1 Department of Pharmacy, University “G. d’Annunzio” Chieti-Pescara, Via dei Vestini 31, 66100 Chieti, Italy; paola.difermo@unich.it (P.D.F.); silvia.dilodovico@unich.it (S.D.L.); rosa.amoroso@unich.it (R.A.); barbara.defilippis@unich.it (B.D.F.); e.dicampli@unich.it (E.D.C.); mara.digiulio@unich.it (M.D.G.); 2 Department of Medical Oral and Biotechnological Sciences, University “G. d’Annunzio” Chieti-Pescara, Via dei Vestini 31, 66100 Chieti, Italy; simonetta.dercole@unich.it

**Keywords:** *Helicobacter pylori* resistance, resveratrol, resveratrol phenol derivatives, antibacterial and anti-virulence action, *Galleria mellonella* model

## Abstract

The drug-resistance phenomenon in *Helicobacter pylori* underlines the need of novel strategies to improve the eradication rate including alternative treatments combining antibiotic and non-antibiotic compounds with synergistic action. In this study, the antibacterial (MIC/MBC) and anti-virulence effects (biofilm reduction and swarming motility inhibition) of resveratrol-RSV and new synthetized RSV-phenol derivatives, with a higher bioavailability, alone and combined with levofloxacin-LVX were evaluated against resistant *H. pylori* clinical strains. The experiments were confirmed in vivo using the *Galleria mellonella* model. Among the studied RSV derivatives, RSV-3 and RSV-4 possessed higher antibacterial activity with respect to RSV (MICs from 6.25 to 200 µg/mL and from 3.12 to 200 µg/mL, respectively). RSV, RSV-3, and RSV-4 were able to synergize with LVX restoring its effect in two out of seven clinical resistant strains tested for the study. RSV, RSV-3, and RSV-4, alone and with LVX at sub-MIC and sub-synergistic concentrations, significantly reduced the biofilm formation. Moreover, RSV-3 and RSV-4 reduced the *H. pylori* swarming motility on soft agar. RSV, RSV-3, and RSV-4 were non-toxic for *G. mellonella* larvae and displayed a protective effect against *H. pylori* infection. Overall, RSV–phenol derivatives should be considered interesting candidates for innovative therapeutic schemes to tackle the *H. pylori* antibiotic resistance.

## 1. Introduction

*Helicobacter pylori* is a gastroduodenal pathogen that affects more than 60% of the population worldwide with a higher prevalence in developing countries [1]. This bacterium is able to colonize the human stomach, thereby inducing inflammation of the gastric mucosa causing chronic or atrophic gastritis, peptic ulcer, gastric mucosa-associated lymphoid tissue (MALT) lymphoma, and gastric cancer [2,3]. The treatment of *H. pylori* infection includes different combinations of drugs among clarithromycin, levofloxacin, amoxicillin, metronidazole, tetracycline, and proton pump inhibitor with an eradication rate that is sometimes satisfactory. The increase of *H. pylori* antimicrobial resistance and the failure of therapeutic schemes underlines the difficulty to treat the *H. pylori* infection [2]. In particular, in our region (Abruzzo region), the resistance of *H. pylori* isolates to clarithromycin is particularly high with values near 73% [4,5].

In the last two decades, the alarming antibiotic resistance phenomenon became a key factor in the treatment failure of *H. pylori* infection, and it strongly suggests us to improve the eradication rate performing susceptibility tests as well as to investigate novel strategies to improve the current therapeutic schemes [4,5,6,7].

*H. pylori* strains, together with genotypic resistance, are also able to express survival strategies entering the dormant state and forming biofilm [8,9,10,11,12]. These antibiotic tolerant conditions widely contribute to the treatment failure and recurrence of infection becoming a significant threat to public health. In fact, *H. pylori* has been included by WHO in the list of pathogens for which identification of novel treatment strategies is urgent.

Recent literature displays the synergistic effect of non-antibiotic compounds when combined with standard therapies with the aim to restore the antimicrobial drug efficacy [13,14,15,16]. This hopeful scenario could be related to the manifold effect on different target sites on the bacterial cell, pharmacokinetic or physicochemical effects (e.g., improvement of solubility or bioavailability), and/or action on a bacterial resistance mechanism [17,18]. Some of our recent studies emphasize the combined synergistic effect between antibiotics and non-antibiotic compounds, resulting in a potentiated effect against *H. pylori* strains [4,13]. In particular, the *Pistacia vera* L. oleoresin or bovine lactoferrin capability to synergize, in vitro and in vivo, with levofloxacin (LVX) by reducing its antimicrobial concentration under the breakpoint, was demonstrated.

**Figure 1:**
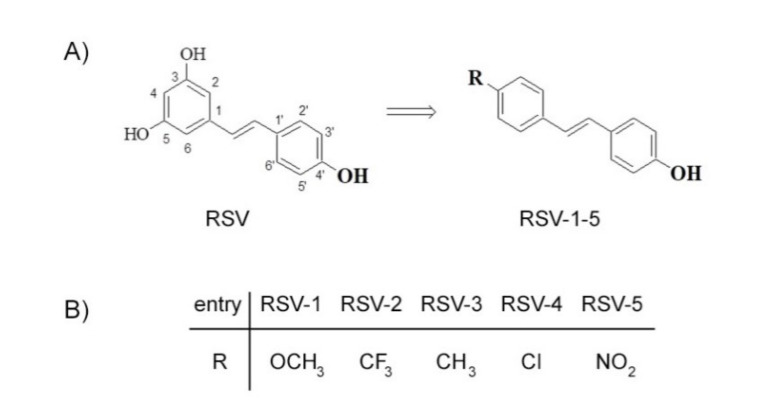
Resveratrol (RSV) (**A**) and its derivatives RSV-1–5 (**B**).

Over recent years, resveratrol (RSV) attracted great attention for its multifaceted biological activities like anti-inflammatory, anti-carcinogenesis, and anti-aging, including antimicrobial activity [19]. Resveratrol is the 3,5,4′-trihydroxy-trans-stilbene (Figure 1A), which is a naturally occurring compound, found, in particular, in grape and in grapeskin, acting like a phytoalexin synthetized in response to microbial attack [20,21]. Resveratrol inhibits the growth of bacteria including both Gram-positive and Gram-negative microorganisms. Among antimicrobial properties [22,23], RSV is able to inhibit ATP synthesis and ATP hydrolysis in *Escherichia coli* and *Mycobacterium smegmatis* [24,25]. This phenolic compound produces, in *E. coli* cells, DNA fragmentation and upregulation of the SOS stress-response regulon, together with a morphological transformation in elongated cells due to the suppression of *fts*Z gene expression. Moreover, RSV displays anti-virulence properties such as antibiofilm, antimotility activities, and it also alters the bacterial exotoxin expression and the quorum sensing system [26,27,28,29,30]. The role of RSV in effecting the oxidative stress and inflammation in *H. pylori*-infected mucosa has also been described [23].

Although RSV possesses antimicrobial benefits, it is noteworthy that its availability is limited. In fact, it is only produced on a nanogram scale in plants, and, thus, it is hardly to be obtained in large quantities from their natural sources. In addition, RSV has been associated with poor bioavailability (less than 1%) especially due to its extensive metabolism [31,32]. To overcome this limitation, various optimization approaches have been developed and RSV was the precursor of more active derivatives, obtained by chemical modification of a stilbene scaffold [33,34]. This approach fits into a context regarding the use of natural products as a chemical lead for development of antibacterial agents [35,36,37,38]. In a recent work, we reported the effects of a series of RSV analogs on viability of three pancreatic cancer cell lines [36]. In order to enlarge the pharmacological activities of these compounds, the interest was to explore the antimicrobial activity against *H. pylori*. For this purpose, among the studied RSV derivatives, we selected the derivatives RSV-1–5 (Figure 1B). They kept the 4′-hydroxyl group because its importance for biological activity has been largely reported [39] while the 3,5-hydroxy group was replaced with a substituent in the 4-position.

In the first step of this study, we evaluated the antibacterial effect of RSV and new synthetized derivatives, RSV-1–5, against resistant *H. pylori* clinical strains. Subsequently, the most antibacterial derivatives were studied for their antimicrobial and anti-virulence effects also combined with LVX, which is an antibiotic commonly used in *H. pylori* therapy. In detail, (i) RSV and the most antibacterial derivatives were combined with LVX for detecting a potential restorative effect in resistant *H. pylori* strains, and (ii) RSV and the most promising antibacterial derivatives were detected for their anti-virulence activity by evaluating the inhibition of biofilm formation alone or combined with LVX and through the swarming motility. The detected in vitro antimicrobial data was confirmed in vivo by using the *Galleria mellonella* model.

The main aim of this work was to explore the potential capability of these novel compounds to synergize with LVX by reducing the level of bacterial persistence/tolerance.

## 2. Results

This study evaluates the antibacterial and anti-virulence properties of RSV and new RSV derivatives and their capability to potentiate the LVX antibacterial action.

The antibacterial effects of RSV and RSV derivatives (1–5) were evaluated against resistant *H. pylori* clinical isolates in terms of MIC and MBC determination (Table 1). As shown, the MIC values of RSV ranged from 200 to 800 μg/mL, among the tested RSV derivatives, with the best promising compounds in terms of antibacterial activity being RSV-3 and RSV-4 with MIC values ranging from 6.25 to 200 μg/mL and from 3.12 to 200 μg/mL, respectively. The detected MBC values were generally one-step or two-step higher than MIC values for RSV and each RSV derivative.

Since RSV-3 and RSV-4 resulted in the most antibacterial compounds with higher antibacterial activity, they were selected together with the lead compound to detect their synergism with LVX, their antibiofilm activity combined with LVX, and their action on *H. pylori* swarming motility.

In Table 2, the best combinations of RSV, RSV-3, or RSV-4 and LVX with the values of FIC Index (FIC I) for all resistant *H. pylori* clinical strains are shown. Synergisms were recorded in *H. pylori* 7A/12 and *H. pylori* 13A/13 with FIC I from 0.24 to 0.28 for *H. pylori* 7A/12 and from 0.26 to 0.28 for *H. pylori* 13A/13. For these two micro-organisms, the LVX MICs, in the presence of RSV, RSV-3, and RSV-4, were reduced from two to eight times by restoring its antimicrobial efficacy. Additive actions were recorded for *H. pylori* 11F/11, 2A/12, 12A/12, 5A/13, and 26A/13. The antagonism was not recorded.

**Figure 2:**
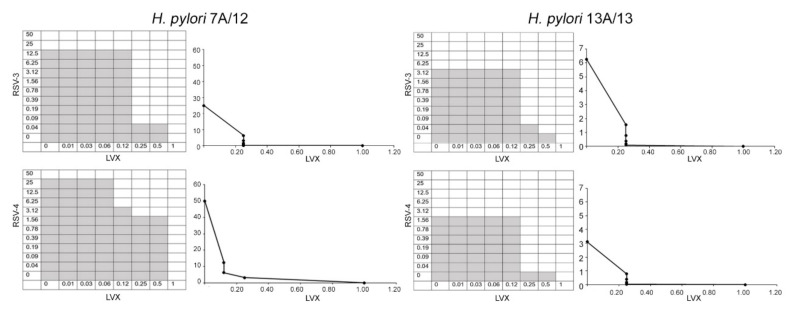
Checkerboard assay and isobolograms showing the synergism between RSV, RSV-3, or RSV-4 with LVX against resistant *H. pylori* 7A/12 and MDR *H. pylori* 13A/13 strains. On the left, representative checkerboard assays, the grey zone represents the bacterial growth and the white zone represents the growth inhibition in the presence of both RSV-3, RSV-4, and LVX. On the right, the isobolograms illustrate the related synergistic curves. The *x*-axis represents the dose of LVX and the *y*-axis represents the dose of RSV-3 and RSV-4. The imaginary straight line connecting the intercept points represents no interaction. Between this line and the synergistic curve, there is the synergistic area (FIC I ≤ 0.5) and additive area (FIC I > 0.5–4.0) interactions. Values above the straight line represent antagonistic interactions (FIC I ≥ 4.0).

Figure 2 shows representative images of checkerboard assays and the respective isobolograms combining different concentrations of RSV-3 or RSV-4 and LVX against two strains: the resistant *H. pylori* 7A/12 and the MDR *H. pylori* 13A/13. RSV, RSV-3, and RSV-4 are able to restore the efficacy of LVX in the *H. pylori* strains.

To evaluate the anti-virulence action of RSV, RSV-3, and RSV-4, the anti-biofilm effects of sub-MIC concentrations of RSV, RSV-3, and RSV-4 alone and combined with LVX at sub-synergistic concentrations were also evaluated for the resistant *H. pylori* 7A/12 and the MDR *H. pylori* 13A/13 for which the LVX restoring effect was observed.

**Figure 3:**
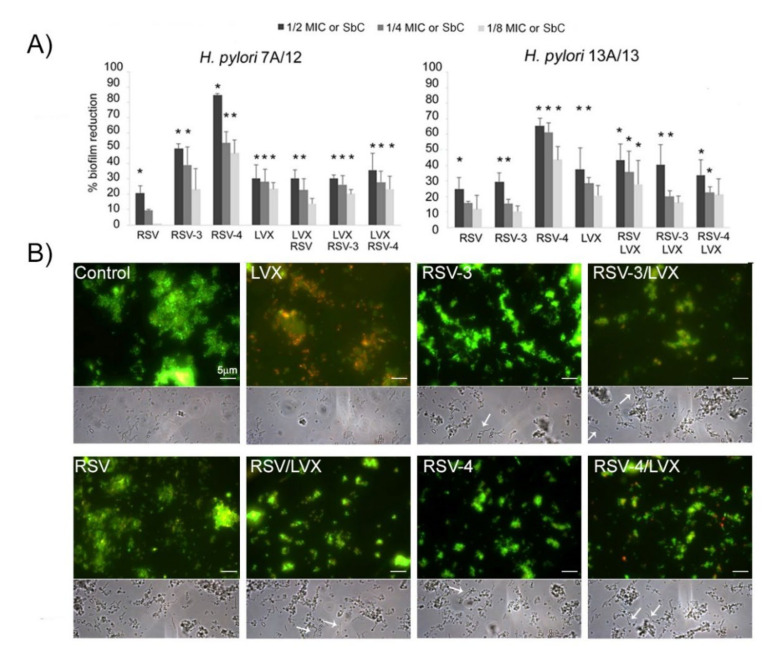
Effect of RSV, its derivatives (RSV-3 and RSV-4), and LVX at sub-inhibitory concentration (1/2, 1/4, 1/8MIC) and their combinations at sub-synergistic concentrations (SbC), against *H. pylori* 7A/12 and *H. pylori* 13A/13 biofilm formation. (**A**) Percentage of biofilm reduction of resistant strain 7A/12 and MDR *H. pylori* 13A/13 after treatments. * Statistically significant values with respect to the control. (**B**) Representative fluorescence (after Live/Dead staining) and phase contrast light microscopy images of *H. pylori* 13A/13 biofilm treated with 1/4 MIC LVX, 1/4 MIC RSV, 1/4 MIC RSV-3, and 1/4 MIC RSV-4 and 1/4 sub-synergistic combinations of RSV, RSV-3, or RSV-4 and LVX and the untreated sample (control). Viable cells exhibit green fluorescence while dead cells exhibit red fluorescence. Arrows indicate the elongated forms of *H. pylori* cells after treatment with RSV, RSV-3, and RSV-4 alone and combined with LVX. Original magnification 1000× (scale bar: 5 μm).

As shown in Figure 3A, for *H. pylori* 7A/12, RSV induced a moderate biofilm reduction not exceeding 20.8% ± 3.9 (1/2MIC). Higher reduction rates were induced by RSV-3 and RSV-4. In particular, RSV-4 produced the highest biofilm inhibition rates with 84.8% ± 0.9, 53.4% ± 7.1, and 46.7% ± 8.4 at 1/2, 1/4, and 1/8 MIC, respectively, with respect to the control (*p* ≤ 0.05). RSV-3 was able to reduce the biofilm formation with a reduction rate of 49.6% ± 3.3, 39.2% ± 11.4, and 23.2% ± 13.3 at 1/2, ¼, and 1/8 MIC, respectively. Regarding the sub-synergistic concentrations, the reduction trend in biofilm formation for RSV, RSV-3, or RSV-4 with LVX was similar (from 30.2% ± 2.2 to 20.3% ± 2.7 for RSV-3 and LVX, from 35.6% ± 11.3 to 23.3% ± 8 for RSV-4 and LVX and from 30.1% ± 5.5 to 13.8% ± 3.3 for RSV and LVX).

For *H. pylori* 13A/13, as shown in Figure 3A, RSV induced a moderate biofilm reduction with a percentage of a biofilm reduction up to 24.8% ± 7.0 (1/2MIC). Similarly to the behaviour toward *H. pylori* 7A/12, RSV-4 expressed the highest *H. pylori* 13A/13 biofilm inhibition rate with respect to the control (65.5% ± 4.0, 61.4% ± 5.0, and 43.7% ± 8.0 at 1/2, 1/4, and 1/8 MIC). Regarding the sub-synergistic concentrations, the reduction trend in biofilm formation for RSV, RSV-3, or RSV-4 with LVX was similar, with a higher percentage of biofilm formation reduction at 1/2 sub-inhibitory concentration (Figure 3A).

Representative Live/Dead staining (Figure 3B, up) and phase contrast (down) images of multidrug-resistant (MDR) *H. pylori* 13A/13 biofilm after treatment with 1/4 MIC LVX, 1/4 MIC RSV, 1/4 MIC RSV-3, and 1/4 MIC RSV-4 and 1/4 sub-synergistic combinations of RSV, RSV-3, or RSV-4 and LVX. A general analysis of the detected microbial biofilms revealed a modification on the aggregation and compactness of treated biofilms with respect to the control through viable staining. This anti-adhesive effect was more pronounced in the presence of RSV-4 and RSV-4 plus LVX. Moreover, samples treated with LVX showed a large amount of red (dead) cells whereas, in all other conditions, green (live) cells were detected underlying the bacteriostatic effect of RSV, RSV-3, and RSV-4.

Micrographs in a phase contrast showed the interesting effect of RSV, RSV-3, and RSV-4 and their combination with LVX in terms of the elongated forms’ presence (Figure 3B, down).

**Figure 4:**
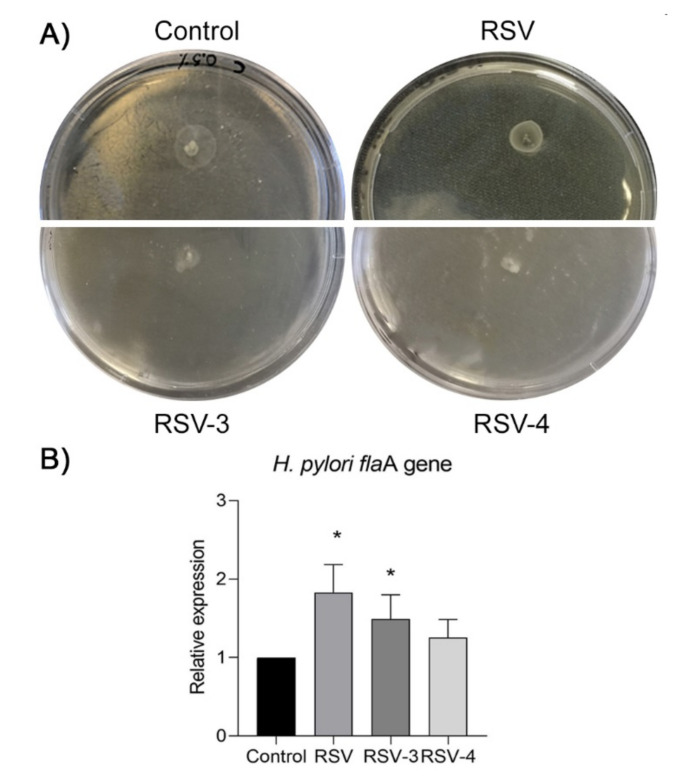
Effect of RSV, RSV-3, and RSV-4 at 1/4 MIC concentrations on the *H. pylori* motility and on the expression of *fla*A gene. (**A**) Representative images of *H. pylori* 13A/13 motility on soft agar 0.5%. RSV-3 and RSV-4 induced a significant loss of motility with respect to RSV and the untreated sample (control), as shown by a smaller diameter of growth on soft agar plates. (**B**) Relative gene expression of *H. pylori fla*A gene in the presence of RSV, RSV-3, and RSV-4. * Statistically significant values with respect to the control.

The soft agar motility assay was performed to evaluate the effect of RSV, RSV-3, and RSV-4 on *H. pylori* swarming motility. For *H. pylori* 7A/12 and *H. pylori* 13A/13, the RSV-3 and RSV-4, included in the soft agar at 1/4 MIC and 1/8 MIC, induced the loss of motility by a smaller diameter of growth in comparison with the control and RSV (*p* < 0.05). Figure 4A shows representative images regarding the loss of *H. pylori* 13A/13 motility in the presence of RSV-3 and RSV-4 at 1/4 MIC concentration. The mean halo diameters for the two analysed strains were 15 mm ± 2 mm for the control, 13 mm ± 2 mm in the presence of 1/4 MIC RSV, 8 mm ± 0.5 mm in the presence of 1/4 MIC RSV-3, and 5 mm ± 1 mm in the presence of 1/4 MIC RSV-4.

In order to clarify the effect of RSV, RSV-3, and RSV-4 on *H. pylori* motility, the expression of the *fla*A gene was performed, which represents the predominant subunits subtype of *H. pylori* flagellum. The expression of *fla*A was induced with response to RSV, RSV-3, RSV-4, and control. *Fla*A gene expression, in the two analysed strains, was significantly increased by RSV (1.86 ± 0.43, fold change) and RSV-3 (1.62 ± 0.31, fold change), (*p* < 0.05) and slightly increased by RSV-4 (1.25 ± 0.28, fold change), (Figure 4B) in apparent contrast with the detected swarming motility.

**Figure 5:**
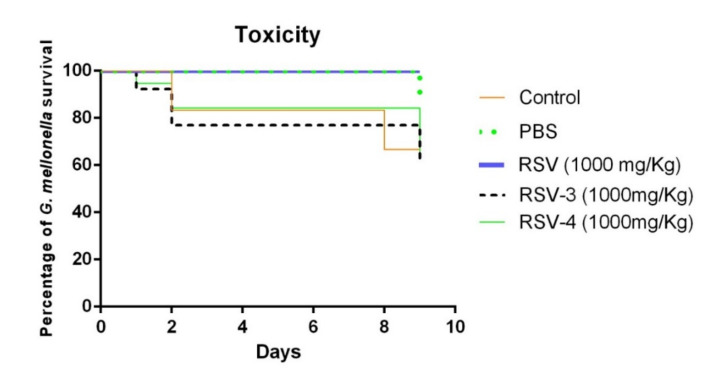
Survival of *Galleria mellonella* larvae in the presence of PBS, RSV, RSV-3, RSV-4 for 9 days, for the toxicity evaluation.

The RSV, RSV-3, and RSV-4 toxicity was evaluated in vivo in *G. mellonella* model. The *G. mellonella* survival percentage at 1000 mg/kg of RSV was 100% after 9 days. For RSV-3, the survival percentage was 90% after 1 day, 80% after 2 days, and 70% after 9 days. A similar RSV-3 trend of survival rate was recorded for RSV-4 with a lower survival rate after 9 days (60%). The survival percentages of *G. mellonella* larvae of the control group and treated with PBS until 8 days were 80% and 100%, respectively (Figure 5). Although the percentage of *G. mellonella* survival, after RSV-3 and RSV-4 treatments, was lower than the RSV treatment, RSV-3 and RSV-4 can be considered non-toxic [40].

**Figure 6:**
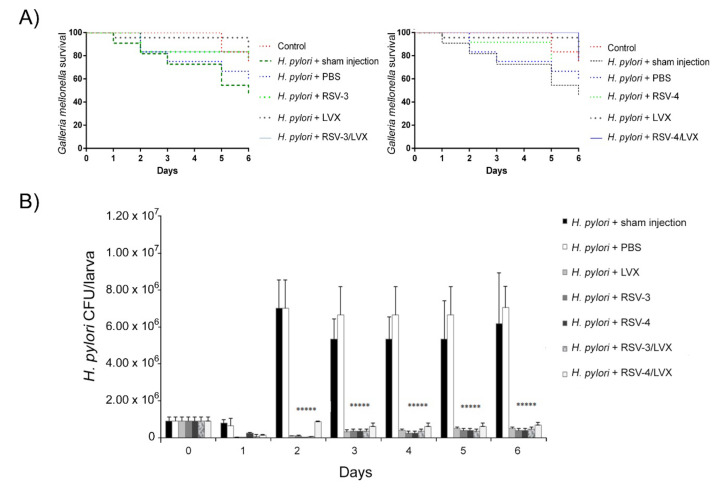
In Vivo infection assay in *Galleria mellonella* larvae. (**A**) Kaplan Meyer survival curves of *G. mellonella* infected with *H. pylori* 13A/13 strain for 2 h, then treated with PBS, LVX, RSV-3, RSV-4, and LVX+RSV-3, LVX+RSV-4 at the best synergistic combination. (**B**) Recovery of *H. pylori* 13A/13 CFU/larva in *G. mellonella* at different time points and different conditions (sham injection, PBS, LVX, RSV-3, and RSV-4 and the best synergistic combination of RSV-3/LVX, RSV-4/LVX). ***** Statistically significant values with respect to the control.

Regarding the *G. mellonella* in vivo infection assay, the survival rate of infected larvae and treated with MIC LVX, RSV-3, and RSV-4, and the best combination of RSV-3 or RSV-4 and LVX (0.09 μg/mL RSV-3 or RSV-4 + 0.25 μg/mL LVX) was monitored every day until the sixth day. After *H. pylori* infection, the treatment with LVX rescued larvae injected with a survival rate between 92% and 75% until 6 days. The treatment with RSV-3 rescued larvae from *H. pylori* infection with 83% of survival until 2 days and 75% at 6 days. The best synergistic combination of RSV-3 plus LVX showed a protective effect against *H. pylori* infection with a larvae survival rate of 100% after 1 day, 83% after 2 days, and 75% after 6 days (Figure 6A). The differences were compared with a Long-rank test and the survival curves were statistically significant.

For RSV-4, the treatment rescued larvae from *H. pylori* infection with an 83% of survival rate after 2 days and 67% after 6 days. The best synergistic combination of RSV-4 plus LVX showed a protective effect against *H. pylori* infection with a larvae survival rate of 100% until 5 days, 75% at 6 days.

RSV-3 and RSV-4 showed in vivo protective effects against an *H. pylori* infection over time, alone and combined with LVX.

The *H. pylori* ability to infect *G. mellonella* larvae was analyzed over time by CFU determination. As shown in Figure 6B, *H. pylori* 13A/13 was able to infect *G. mellonella* larvae and grow over time. In the presence of LVX, the high survival rate of *G. mellonella* larvae was related to the reduced *H. pylori* survival rate, shown by the lower CFU values detected with respect to the sham injection and PBS treatment (reduction in CFUs of 98% with respect to sham injection and 94% with respect to treatment with PBS) after 5 days (*p* ≤ 0.05). RSV-3 and RSV-4 showed their protective effect in *G. mellonella* larvae reducing significantly the *H. pylori* CFUs of 97.2% with respect to sham injection and 92.4% with respect to PBS treatment for 5 days for both derivatives. In the presence of the best synergistic combinations RSV-3 or RSV-4 and LVX, an interesting *H. pylori* CFU/larva reduction with 92.4% (for RSV-3 and LVX) and 90% (for RSV-4 and LVX) until 5 days (*p* ≤ 0.05) was detected (Figure 6B).

## 3. Discussion

Resveratrol is a naturally occurring polyphenolic antioxidant, belonging to the stilbene family, produced by the plants in response to a microbial attack. The antibacterial activity of RSV has been described, based on more than a single mechanism, such as alteration of the lipid bilayer of the membrane and its permeability, changes in intracellular functions induced by hydrogen bonding of the phenolic group in 4-position to enzymes, and antioxidant-scavenging activity that could inhibit the generation of reactive oxygen species, reducing the redox potential of the growth medium [41,42,43,44].

The antibacterial and anti-virulence activities of RSV and newly synthetized RSV derivatives, chosen to overcome the limitation of the poor bioavailability of RSV combined with LVX against resistant *H. pylori* clinical strains, were investigated.

Our results show an antimicrobial activity of RSV against resistant *H. pylori* strains similar to previous studies [45,46,47,48,49]. In particular, Paulo et al. [28] suggested a possible mechanism of RSV action related to the inhibition of *H. pylori* urease activity, preventing the production of the alkaline environment around bacterial cells, allowing the microorganism to survive the stomach acidic conditions. However, RSV seems to be less active against Gram-negative bacteria than Gram-positive. Likely, the presence of efflux pump systems in Gram-negative may be responsible for their decreasing susceptibility to this natural stilbenoid, as documented by experiments performed with mutants or in the presence of efflux pumps inhibitors [50,51,52]. This observation leads us to hypothesize that the antibacterial action of RSV could be due to its interaction with targets present inside cytoplasmic or in periplasmic sites in Gram-negative bacteria [19].

As known, RSV shows low bioavailability so, in this work, newly synthetized RSV derivatives were investigated for their antibacterial properties. To this aim, selected derivatives 1–5 were chosen. They kept the 4′-hydroxyl group because its importance for biological activity and the 3,5-hydroxy group was replaced with a substituent in the 4-position.

Regarding the newly synthetized RSV derivatives, the RSV-3 (R = Me) and RSV-4 (R = Cl) showed the best antibacterial action at very low concentrations. These RSV derivatives show greater effectiveness than the lead compound, RSV. The MIC of RSV-4 was 64-fold lower than RSV against the *H. pylori* strains, suggesting the improved antibacterial activity in RSV derivatives. In compounds RSV-3 and RSV-4, the 3,5-dihydroxy motif of RSV was substituted with a more lipophilic methyl (RSV-3) and chlorine (RSV-4), keeping the unchanged OH-group in the 4′-position. It can be hypothesized that, in this way, the mechanism of antimicrobial action of RSV remains unchanged due to the presence of the OH-group in the 4′-position. However, the loss of two hydrophilic OH-groups and the introduction of a lipophilic group could be beneficial for overcoming the cell membrane in a passive way and inducing antibacterial action inside cells. Likely, the simpler and more lipophilic structures of RSV-3 and RSV-4 could promote their entry into the bacterial cell and induce its antibacterial action inside the cells. In order to elucidate the possible mechanism of action, further studies are required.

The capability of RSV, RSV-3, and RSV-4 to synergize with LVX to restore the antibiotic efficacy and reduce its MIC values under the breakpoint was also evaluated. To the best of our knowledge, this is the first study in which the capability of RSV and RSV derivatives to synergize with antibiotics was evaluated. Xia et al. [53] showed the RSV synergism when combined with alcohol, underlying that the anti-*H. pylori* mechanism of RSV is linked to its inhibitory effect on translation, outer membrane protein production, ATP synthase, and transports.

To explore the anti-virulence activities of RSV, RSV-3, and RSV-4, we evaluated their effect on biofilm formation and *H. pylori* motility.

Regarding the effect on microbial biofilm formation, RSV, RSV-3, and RSV-4 were detected alone and in combination with LVX at sub-synergistic concentrations. Our results highlighted a moderate RSV antibiofilm effect on resistant and MDR *H. pylori* strains. RSV-3 and RSV-4 exhibited a higher antibiofilm activity, likely due to their higher bioavailability and ability to interact with the bacterial cells. It was proposed that inducing the down regulation of outer membrane proteins, RSV, and likely RSV-3 and RSV-4 affect the bacterial adhesion and colonization [52,54]. A study by Klancnik et al. [55] evaluated the effect of RSV on the biofilm formation in *Campylobacter jejuni* together with the culturability and viability of the adhered cells post-treatment with RSV. Sub-inhibitory concentration of RSV induced 40% inhibition of *C. jejuni* biofilm formation interfering in the quorum sensing system [55].

Regarding the anti-virulence activity of RSV, RSV-3, and RSV-4, we evaluated their effect on *H. pylori* motility. As well known, the flagellar motility represents for *H. pylori* an important virulence factor for successful colonization in vivo of gastric mucosa, for chemotaxis, and for its movement toward the gastric mucus within the stomach [51]. From our results, on soft agar, it was possible to observe the loss of *H. pylori* motility in the presence of RSV-3 and RSV-4 at sub-MIC concentrations and a moderate anti-motility action in the presence of RSV. As reported by others [55,56,57], at sub-inhibitory concentrations, RSV reduced the swarming motility in a dose-dependent manner in *Proteus mirabilis,* the swimming and swarming motility in *E. coli* as well as in *Vibrio vulnificus*.

In this work, RSV, RSV-3, and, to a lesser extent, RSV-4, induced the over-expression of the *flaA* gene. This result could seem an apparent contradiction when looking the phenotypic motility on a soft agar plate. Since the control system for the flagellar motility in *H. pylori* is an intricate process not dependent only on FlaA production [58,59] and considering that, in *E. coli* and *M. smegmatis,* RSV is able to inhibit ATP synthesis [24,28]. We can hypothesize that RSV and RSV derivatives, affecting the flagellar hook and its energy production system could compromise the flagellar functionality. We can suppose that *H. pylori* cells, in the presence of RSV, RSV-3, or RSV-4 are characterised by a major production of flagella but their efficacy is reduced by an insufficient ATP synthesis system resulting in a reduced swarming motility. More studies are required to explore this complex mechanism.

RSV, RSV-3, and RSV-4 were able to induce an elongated morphology in *H. pylori* cells, as reported, for RSV, in other Gram negative [29,30]. This atypical filamentous morphology might be due to an alteration during the regular cell division and to the FtsZ ring uncorrected production compromising the microbial division.

Moreover, infecting *G. mellonella* larvae with *H. pylori* MDR strain and treating, then, with RSV-3 and RSV-4 alone and combined with LVX at the best synergistic combinations, an interesting protective effect against *H. pylori* was observed, confirming the in vitro results. The proven effectiveness of these compounds, in the in vivo model, allow us to emphasize the RSV derivatives’ possible role in the management of *H. pylori* infection.

Despite the limitation of this study related to a few numbers of resistant *H. pylori* detected micro-organisms, we can conclude that, on the basis of the interesting antibacterial action, and their effects on biofilm formation and on bacterial motility, RSV–phenol derivatives could be considered interesting anti-*H. pylori* candidates for innovative, therapeutic schemes to tackle the *H. pylori* antibiotic resistance.

## 4. Materials and Methods

### 4.1. Chemistry

The synthesis of compounds RSV-1–5 was carried out as previously reported [36,37].

The 4-hydroxybenzaldehyde and the appropriate aryl acetic acid were mixed in the presence of piperidine at 130°C. The usual aqueous work-up and purification using silica gel column chromatography produced the desired phenols. The chemical stability of all compounds was evaluated before and after the sterilization in autoclave (Fedegari Autoclavi S.p.a., Albuzzano (PV), Italy) comparing the ^1^H-NMR signals on a Varian Mercury 300 spectrometer (Varian, Palo Alto, CA, USA).

### 4.2. Bacterial Cultures

Seven clinical *H. pylori* isolated strains, coming from the private collection of Bacteriological Laboratory of the Pharmacy Department, University “G. d’Annunzio” Chieti-Pescara, were used in the experiments. Strains were previously characterized for their antimicrobial susceptibility profiles against the antibiotic commonly used in therapy: clarithromycin, metronidazole, levofloxacin (LVX), moxifloxacin, ciprofloxacin, rifabutin, tetracycline, ampicillin, and amoxicillin (Table S1) [13]. All strains were resistant to LVX (*H. pylori* 2A/12 was also included despite its borderline MIC LVX values between 0.5–1.00 μg/mL). *Helicobacter pylori* 11F/11, *H. pylori* 2A/12, and *H. pylori* 7A/12 were resistant to at least three antibiotics, whereas *H. pylori* 5A/13, *H. pylori* 13A/13, and *H. pylori* 26A/13 were multi-drug resistant (MDR) strains with resistance profiles of at least three antimicrobial classes. The reference strain *H*. *pylori* ATCC 43629 was also included. The study was approved by the Inter Institutional Ethic Committee of University “G. d’Annunzio” Chieti-Pescara, Chieti, Italy (ID Number RICH9RTLH). Bacteria were cultured on Columbia agar base (CA, Oxoid, Milan, Italy) with 10% (*v*/*v*) lacked horse blood plus IsoVitalex 1% (*v*/*v*) (BBL, Microbiology System, Milan, Italy). For the experiments, the bacterial suspensions were prepared in Brucella Broth (BB) plus 2% foetal calf serum (FS) (Biolife Italiana, Milan, Italy) and adjusted to an optical density at 600 nm (OD_600_) of 0.2 corresponding to ~1.8 × 10^6^ Colony Forming Unit (CFU)/mL

### 4.3. Antibacterial Susceptibility Assay

The determination of Minimum Inhibitory Concentration (MIC) values of RSV and new synthesized RSV-1–5 against standardized broth cultures of *H*. *pylori* strains was performed by a microdilution method assay in 96-wells-microtitre plates (Nunc, Euro Clone SpA, Life Sciences-Division, Milan, Italy). Two-fold dilutions of RSV and RSV-1–5 stock solution, ranging from 800 to 6.25 μg/mL, were prepared in BB plus 2% FS. Levofloxacin (Sigma Aldrich S.R.L, Milan, Italy) was prepared in BB plus 2% FS in two-fold dilutions from 2.00 to 0.01 μg/mL. One hundred μL of RSV or each RSV derivative or 100 μL LVX and 100 μL of standardized bacteria were dispensed in each well of 96-wells-microtitre plate and incubated in micro-aerobic condition (5% O_2_, 10% CO_2_, 85% N_2_) for 3 days at 37 °C.

MIC values were measured by determining the lowest concentration of substances able to inhibit the visible growth of the microorganisms. Minimum Bactericidal Concentrations (MBCs) were determined by sub-cultivation of 10 μL of suspensions from the non-turbid wells on CA and incubated as describe above. The MBC represents the lowest concentration of each substance that inhibited the bacterial growth on plates. The MBC values were also confirmed by an iodo-nitro tetrazolium violet assay (INT, Sigma-Aldrich) following the addition (40 μL) of 0.2 mg/mL of INT and incubation at 37 °C for 2 h. Viable bacteria reduce the yellow dye to a pink-purple and dead cells do not produce a color change.

### 4.4. Checkerboard Assay

The checkerboard test was performed to evaluate the synergism between RSV or the most promising antibacterial derivatives (RSV-3 or RSV-4) and LVX against *H. pylori* strains.

Dilutions of the RSV or RSV-3, RSV-4, and LVX from MIC values to serial dilution below were inoculated in 96-well microtiter plates and incubated as described above. The checkerboard test was used to calculate the Fractional Inhibitory Concentration (FIC) equal to MIC_AB_/MIC_A_ + MIC_BA_/MIC_B_, where MIC_AB_ is the MIC of compound A in the presence of compound B. MIC_BA_ is MIC of B in the presence of A. FIC Index (FIC I) values were interpreted according to Odds [60] as follows: synergism FIC I ≤ 0.5, antagonism FIC I ≥ 4.0, and additive FIC I > 0.5–4.0. All studied substances (RSV, RSV-3, RSV-4) and LVX were assayed alone as a control. The results were also reported as isobolograms constructed by plotting synergistic concentrations [13].

### 4.5. Biofilm Biomass Quantification and Microscopic Analysis

The anti-biofilm activity of RSV, RSV-3, and RSV-4 (at sub-MIC values) were evaluated on the biofilm formation of selected *H. pylori* strains: resistant *H. pylori* 7A/12 strain and MDR *H. pylori* 13A/13 strain, toward which the synergistic action with LVX was observed. Broth cultures of *H*. *pylori*, harvested in BB with 2% (*w*/*v*) FS and 0.3% (*w*/*v*) glucose, were gently shaken and incubated overnight at 37 °C in a micro-aerobic atmosphere. After incubation, each broth culture was adjusted to OD_600_~0.2 and 100 μL were inoculated on flat-bottomed 96-wells-polystyrene-microtiter plates with a sub-MIC concentration of RSV or RSV-3-4 (100 μL), LVX (100 μL), and the best synergistic combinations at a sub-synergistic concentration. After incubation at 37 °C in microaerobic condition for 48 h, the produced biomasses of the treated and untreated biofilms were quantified by a safranin staining method [13].

For the cell viability evaluation, biofilms were grown as described above. Briefly, 1 mL of sub-MIC RSV, or sub-MIC of RSV-3, RSV-4, or 1 mL of sub-MIC LVX, or 1 mL of the best synergistic combinations at a sub-synergistic concentration and 1 mL of standardized broth cultures of each *H*. *pylori* strains were inoculated in a Petri dish (3.5 cm) and incubated at 37 °C in a microaerobic condition for 48 h. After incubation, the planktonic phase was removed and the sessile bacterial populations were washed with PBS and stained with Backlight Live/Dead Viability staining (Molecular Probes, Invitrogen detection technologies, USA) as indicated by the manufacturer [61]. The images were observed at Leica 4000 DM fluorescent microscopy (Leica Microsystems, Milan, Italy), and more fields of view were examined randomly [14]. Moreover, the samples were also observed under a phase contrast light microscope (Leica 4000 DM) to observe the *H. pylori* morphology.

### 4.6. Motility and *fla*A Gene Expression Assays

The effect of RSV, RSV-3, and RSV-4 on the resistant *H. pylori* 7A/12 strain and MDR *H. pylori* 13A/13 strain motility was evaluated following the Ciccaglione et al. [4] methodology with some modifications. Briefly, *H. pylori* clinical strains motility was analysed by using soft agar plates composed by BB plus 10% FS, 0.5% agar bacteriological, and sub-MIC concentration (1/4MIC, 1/8MIC) of RSV, RSV-3 and RSV-4. Ten microliters of overnight broth cultures standardized at 600 nm (OD_600_) of 0.4 in BB, plus 10% FS, were inoculated into the thickness of the soft agar with a sterile tip. Plates were incubated at 37 °C under microaerobic conditions for 5–6 days. Then the bacterial halo was recorded.

For the evaluation of *fla*A gene expression, *H. pylori* broth cultures in 40 mL of BB plus 2% FS at OD_600_ ~0.2 were incubated in the presence of 1/4 MIC of RSV or 1/4 MIC of RSV-3 or RSV-4 at 37 °C for 3 days in a micro-aerobic condition. Subsequently, *H. pylori* cells were harvested by centrifugation at 5000× *g* for 10 min at 4 °C and washed three times with sterile PBS.

Total RNA extraction was performed using the RNeasy mini kit (Qiagen, Milan, Italy), according to the manufacturer’s instructions. cDNA was generated using the iScript cDNA Synthesis Kit (Bio-Rad, Milan, Italy) and then stored at −20 °C until use. For the quantitative PCR, the oligonucleotide primers used were the *fla*A gene sequence Fwd: 5′-CAGTATAGATGGTCGTGGGATTG-3′, Rvs: 5′-GAGAGAAAGCCTTCCGTAGTTAG-3′; the housekeeping gene 16SrRNA Fwd: 5′-GGAGTACGGTCGCAAGATTAAA-3′ Rsv: 5′-CTAGCGGATTCTCTCAATGTCAA-3′ [62]. (The primers showed ≥ 95% efficiency values). The quantitative PCR reactions were performed in 96-well microtiter plates (Bio-Rad, Milan, Italy) using 10 μL of SsoAdvanced universal SYBR Green supermix 2X (Bio-Rad, Milan, Italy), 0.6 μM forward/reverse primer mix, and 2 μL of cDNA, in free-nucleases water to a final volume of 20 μL. The qPCR reactions were placed into a CFX96 Real Time system, C1000 Touch, Thermal Cycler (Bio-Rad, Milan, Italy).

Thermal cycling conditions were as follows: 3 min at 95 °C for initial denaturation followed by 39 cycles of 95 °C for 15 s and 55 °C for 40 s. After a finalization step of 15 s at 95 °C and 55 °C for 40 s, a melting curve analysis was performed with a temperature range between 65 °C and 95 °C and an increment of 0.5 °C for 15 s followed by the plate read out. A melting curve was used at the end to confirm only one peak and only one product. The values of the threshold cycle (Ct) and relative expression level were normalized by the ΔΔCT method. Results were analysed using the Bio-Rad CFX Manager Software, version 3.1 (Bio-Rad Laboratories).

### 4.7. Toxicity Test in the Galleria mellonella Model

The toxicity of RSV, RSV-3, and RSV-4 was evaluated by using wax moth *G*. *mellonella* larvae. Stock solutions of RSV, RSV-3, and RSV-4 were diluted in PBS to obtain the final concentration of 1000 mg/Kg. Five groups of 10 randomly-selected *G*. *mellonella* larvae, choosing with a weight ranging between 0.2–0.3 g, were treated as follows: three groups were injected in the last left proleg with 10 μL of RSV, 10 μL of RSV-3, 10 μL of RSV-4, one group was injected with 10 μL of PBS by using 0.3 mL micro-fine needle insulin syringes (BD, Milan, Italy), and one group was un-injected. A total of 50 larvae were incubated at 37 °C in Petri dishes in the dark for nine days. The wax moth survival was monitored over nine days, every 24 h. The larvae were considered dead when they were unresponsive to touch [13]. During assays, larvae did not receive nutrition.

### 4.8. In Vivo G. mellonella Infection Assay

The activity of RSV-3 and RSV-4 against *H*. *pylori* 13A/13 infection was evaluated by the in vivo model of *G*. *mellonella* larvae, which represents a recognized model for *H*. *pylori* infection for which no ethical approval is required. *H*. *pylori* studied strain 13A/13, standardized at OD_600_ = 0.2 (~1.8 × 10^6^ CFU/mL), was chosen for the experiments. Seven groups of 10 randomly selected *G*. *mellonella* larvae were injected with 10 μL of *H*. *pylori* broth culture in the last left proleg of each larva for a total of 70 larvae. One group of 10 larvae was not infected. After 2 h of infection, 10 larvae were treated with 10 μL of LVX at the MIC value, 10 larvae were treated with RSV-3 or RSV-4 at 1000 mg/Kg, and 10 larvae were treated with the best synergistic combination of RSV-3 or RSV-4 plus LVX on the last right proleg. A control group of 10 larvae was treated with 10 μL of PBS and 10 larvae with a sham injection (larvae were nicked with a sterile syringe to evaluate the effect of the larval puncture). *G*. *mellonella* larvae were incubated at 37 °C in the dark for 6 days. During assays, larvae did not receive nutrition. The *G*. *mellonella* survival was controlled every day. Dead larvae were unresponsive to touch.

To evaluate the *H*. *pylori* 13A/13 survival rate in *G*. *mellonella* larvae after 1-, 2-, 3-, 4-, 5-, and 6-days post-infection, seven groups of 10 randomly selected *G*. *mellonella* larvae were infected as described above. After incubation, for each group, three larvae were chilled on ice for 10 min, and the haemocoel was serially diluted in sterile PBS and the *H. pylori* cells were quantified by CFUs determination on Campylobacter selective agar (CP Dent) with 7% defibrinated horse blood and 0.4% of Dent supplement (Oxoid) and incubated in a microaerobic condition at 37 °C. The CFU/larva were counted after 3 days.

### 4.9. Statistical Analysis

Data is obtained from at least three independent experiments performed in triplicate. Data is shown as the means  ±  standard deviation.

Differences between groups were assessed with a paired Student’s *t*-test. *p* values  ≤  0.05 were considered statistically significant. Survival curves were plotted using the Kaplan-Meier method, and survival differences were calculated using the Long-rank test for multiple comparisons. GraphPad Prism 6 was used to fit a curve to the infection data.

## Figures and Tables

**Table 1:** Minimum Inhibitory Concentration (MIC, μg/mL) and Minimum Bactericidal Concentration (MBC, μg/mL) of RSV and RSV derivatives (RSV-1–5) against resistant *H. pylori* strains.

	Substances													
***H. pylori* strains**	**LVX**		**RSV**		**RSV-1**		**RSV-2**		**RSV-3**		**RSV-4**		**RSV-5**	
	MIC	MBC	MIC	MBC	MIC	MBC	MIC	MBC	MIC	MBC	MIC	MBC	MIC	MBC
11F/11	1.00	1.00	200	400	200	800	200	200	25	50	3.12	25	200	400
2A/12	0.50–1.00	1.00	200	200	200	400	200	200	25	25	3.12	50	200	200
7A/12	1.00	1.00	200	200	>800	>800	200	400	25	25	50	50	200	400
12A/12	1.00	1.00	200	200	100	400	400	800	6.25	50	25	100	200	400
5A/13	1.00	1.00	800	>800	200	400	100	200	200	400	200	400	100	100
13A/13	1.00	1.00	200	400	100	400	50	100	6.25	50	3.12	50	100	200
26A/13	2.00	2.00	800	>800	200	400	100	100	50	100	100	200	200	200
ATCC 43629	0.12	0.12	200	800	100	>800	25	100	6.25	50	12.5	25	100	100

**Table 2:** Best combinations (μg/mL) of RSV, RSV-3 and RSV-4 and LVX with the values of FIC Index (FIC I) for resistant *H. pylori* clinical strains.

*H. pylori* Strains	Best Synergistic Combinations					
	LVX+RSV	FIC I	LVX+RSV-3	FIC I	LVX+RSV-4	FIC I
11F/11	1.00+0.50	1.50	1.00+0.09	1.00	1.00+0.01	1.00
2A/12	0.50+0.50	1.00	0.50+0.09	1.00	0.50+0.09	1.03
7A/12	0.25+6.00	0.28	0.25+0.09	0.25	0.12+6.25	0.24
12A/12	1.00+0.50	1.50	1.00+6.25	2.00	1.00+25.0	2.00
5A/13	1.00+24.0	1.03	1.00+0.78	1.00	1.00+0.78	1.00
13A/13	0.25+6.00	0.28	0.25+0.09	0.26	0.25+0.09	0.26
26A/13	2.00+24.0	1.03	2.00+12.5	1.25	2.00+1.25	1.12

## Supplementary Materials

The following are available online at https://www.mdpi.com/2079-6382/9/12/891/s1, Table S1: Antimicrobial susceptibility panel of *H. pylori* clinical strains used in this study.
