# Supplementary material for: Searching for New Tools to Counteract the Helicobacter pylori Resistance: The Positive Action of Resveratrol Derivatives
Source: Antibiotics (Basel). 2020 Dec 10;9(12):891. doi: 10.3390/antibiotics9120891 (PMC7763357; doi:10.3390/antibiotics9120891)
Supplement: Supplementary file 1 [file antibiotics-09-00891-s001.pdf]

# Supplementary Material

## Searching for new tools to counteract the *Helicobacter pylori* resistance: the positive action of resveratrol derivatives

**Paola Di Fermo[1#], Silvia Di Lodovico[1#], Rosa Amoroso[1], Barbara De Filippis[1], Simonetta D'Ercole[2], Emanuela Di Campli[1], Luigina Cellini[1*] and Mara Di Giulio[1]**

[1] Department of Pharmacy, "University "G. d'Annunzio" Chieti-Pescara, Via dei Vestini 31, 66100 Chieti (CH), Italy; paola.difermo@unich.it (P.D.F.); silvia.dilodovico@unich.it (S.D.L.); rosa.amoroso@unich.it (R.A.); barbara.defilippis@unich.it (B.D.F.); e.dicampli@unich.it (E.D.C.); l.cellini@unich.it (L.C.); mara.digiulio@unich.it (M.D.G.)

[2] Department of Medical Oral and Biotechnological Sciences, University "G. d'Annunzio" Chieti-Pescara, Via dei Vestini 31, 66100 Chieti (CH), Italy; simonetta.dercole@unich.it (S.D.E)

* Correspondence: l.cellini@unich.it Tel.: +39-0871-3554560

\# Paola Di Fermo and Silvia Di Lodovico contributed equally to this work

**Table S1** Antimicrobial susceptibility panel of *H. pylori* clinical strains used in this study

| *H. pylori* | Antimicrobials* | | | | | | | | |
|---|---|---|---|---|---|---|---|---|---|
| | CLA | MET | LVX | MOXI | CIP | RIF | TET | AMP | AMOX |
| **11F/11** | R | S | R | R | R | S | S | S | S |
| 2A/12 | R | R | R | R | R | S | S | S | S |
| 7A/12 | S | S | R | R | R | S | S | S | S |
| 12A/12 | R | S | R | R | R | S | S | S | S |
| 5A/13 | R | R | R | R | R | S | S | S | S |
| 13A/13 | R | R | R | R | R | S | S | S | S |
| 26A/13 | R | R | R | R | R | S | S | S | S |
| ATCC 43629 | S | S | S | S | S | S | S | S | S |

*Abbreviations: CLA, clarithromycin; MET, metronidazole; LVX, levofloxacin, MOXI, moxifloxacin; CIP, ciprofloxacin; RIF, rifabutin; TET, tetracycline; AMP, ampicillin; AMOX, amoxicillin.
